# Short- and Long-Term Effects of a Single Exposure to Gadoteric Acid in Healthy Rats—Gadolinium, Iron and Other Metal Accumulation in Kidney and Brain

**DOI:** 10.3390/ijms27146208

**Published:** 2026-07-11

**Authors:** Susana Coimbra, Susana Rocha, Rui Azevedo, Sofia D. Viana, Petronila Rocha-Pereira, Maria João Valente, Cristina Catarino, Luís Belo, Elsa Bronze-da-Rocha, Agostinho Almeida, Flávio Reis, Alice Santos-Silva

**Affiliations:** 1 UCIBIO i4HB, Faculdade de Farmácia, Universidade do Porto, Rua Jorge Viterbo 228, 4050-313 Porto, Portugal; srocha@ff.up.pt (S.R.); petronilarochapereira58@gmail.com (P.R.-P.); mvalente@ff.up.pt (M.J.V.); cristinacatarino@ff.up.pt (C.C.); luisbelo@ff.up.pt (L.B.); elsa.rocha@ff.up.pt (E.B.-d.-R.); 2 UCIBIO i4HB, Translational Toxicology Research Laboratory, University Institute of Health Sciences (1H-TOXRUN, IUCS-CESPU), Avenida Central de Gandra 1317, 4585-116 Gandra, Portugal; 3 LAQV REQUIMTE, Faculdade de Farmácia, Universidade do Porto, Rua de Jorge Viterbo Ferreira 228, 4050-313 Porto, Portugal; ruiazevedo43@gmail.com (R.A.); aalmeida@ff.up.pt (A.A.); 4 Institute of Pharmacology & Experimental Therapeutics, Faculty of Medicine, University of Coimbra, Azinhaga de Santa Comba, Celas, 3000-548 Coimbra, Portugal; sofia.viana@ff.uc.pt (S.D.V.); freis@fmed.uc.pt (F.R.); 5 Coimbra Institute for Clinical and Biomedical Research (iCBR), Faculty of Medicine, University of Coimbra, Azinhaga de Santa Comba, Celas, 3000-548 Coimbra, Portugal; 6 CIBB—Center for Innovative Biomedicine and Biotechnology, University of Coimbra, Rua Larga, Edifício FMUC, Piso 1, 3004-504 Coimbra, Portugal; 7 Faculty of Pharmacy, University of Coimbra, Azinhaga de Santa Comba, Celas, 3000-548 Coimbra, Portugal; 8 Clinical Academic Center of Coimbra (CACC), 3004-561 Coimbra, Portugal

**Keywords:** Gd (III) retention, iron metabolism, metal homeostasis, kidney, brain, GBCA, MRI, chelates, nephrotoxicity

## Abstract

Gadolinium [Gd (III)] released from gadolinium-based contrast agents, commonly used in magnetic resonance imaging, may accumulate in organs, altering metal homeostasis. We aimed to clarify Gd (III), iron and other metal accumulation in the kidney and brain, in the short (2 days) and long term (20 weeks), following exposure to gadoteric acid (Gd-DOTA) or free Gd (III). Wistar rats received one dose of Gd (III), Gd-DOTA, or saline. Gd (III) and metal levels in the blood (whole blood, and serum in the case of iron), kidney, and brain, and iron metabolism biomarkers, were assessed. Gd (III) was detectable in the blood, kidney, and brain, at both time points, for both Gd (III) and Gd-DOTA-treated groups. Exposure to free Gd (III) showed a significant Gd (III) accumulation in the kidney, brain and blood in the short term and long term; Gd-DOTA presented significant accumulation only in the short term. Gd-DOTA appears to have faster elimination and minimal brain deposition. Exposure to free Gd (III), but not to Gd-DOTA, led to disturbances in metal homeostasis and in iron metabolism markers (serum ferritin and transferrin saturation), but did not alter tissue iron levels. In summary, the kidney appears as the primary site of accumulation and retention. Despite the safer profile shown by Gd-DOTA in the long term, our data highlight the importance of clarifying the pathophysiological implications of Gd (III) retention and/or accumulation, particularly in vulnerable conditions and repeated exposures.

## 1. Introduction

Gadolinium [Gd (III)] is a heavy metal suitable for enhancement of magnetic resonance imaging (MRI), which is commonly used for diagnosis, staging, and disease monitoring. Free Gd (III) exerts adverse effects on several organs, disturbing several physiological processes. To minimize free Gd (III) toxicity, it is stabilized by chelation, for use in MRI studies. Even so, exposure to gadolinium-based contrast agents (GBCA) is still associated with adverse effects, targeting different tissues and organs.

The release of Gd (III) from GBCA appears to result from the competition of endogenous bivalent cations, such as iron, zinc, calcium, copper or manganese, for the ligand, replacing Gd (III), a reaction known as transmetallation. Iron, followed by copper and zinc, showed the highest affinities for the ligands [1,2]; however, several factors may influence transmetallation in vivo, namely the physicochemical properties of the GBCA and endogenous physiological factors [1]. It was reported [3] that the release of Gd(III) is greater from nonionic linear GBCA than from ionic linear agents; elevated serum phosphate concentrations accelerate Gd(III) release from both classes of linear GBCA, which was higher for nonionic linear compounds; in contrast, the evaluated macrocyclic GBCA remained stable, regardless of phosphate serum concentration. The dissociated Gd (III) may deposit in multiple organs, namely in the kidneys [4] and brain [5], entailing long-term toxicity, which has been attributed to a slow release of Gd (III) from the deposits into the bloodstream. In agreement with these findings, the use of metal binding agents decreased transmetallation of GBCA [6,7], suggesting that inhibited transmetallation may reduce or prevent Gd (III) retention in tissues.

The kidney is one of the major targets of Gd (III), since renal excretion is the main elimination route for most GBCA. Nephrogenic systemic fibrosis (NSF), a severe renal complication [8], was associated with GBCA administration, especially in patients with renal impairment [9]. In animals without kidney disease, exposure to GBCA led to tubular necrosis [10]. Another study in healthy animals showed that GBCA also caused kidney injury and significant metabolic alterations [11]. More recently, we reported that a single dose of free Gd (III) or of gadoteric acid (Gd-DOTA), a macrocyclic GBCA, in healthy Wistar rats, induced several (hematological, inflammatory, lipid, hepatic and renal) changes 2 days after exposure, which were more prominent for free Gd (III), whereas 20 weeks after exposure, much fewer changes were observed, especially for Gd-DOTA [12]. Accordingly, a study by Rasschaert et al. [13] investigating Gd (III) deposition in several organs of healthy animals following administration of a single dose of macrocyclic GBCA, including Gd-DOTA, at different time points, showed that the kidney presented the highest Gd (III) accumulation.

Animal studies have also shown that the amounts of Gd (III) retained in the brain, bones, skin and other organs are higher for linear GBCA than for those with a macrocyclic structure [1,14,15]. In fact, nowadays, the use of macrocyclic GBCA, more stable chelates of Gd (III), is favored over linear agents, but even these strong chelates have been associated with Gd (III) release and retention in the brain, and with the development of NSF [14,16,17].

Gd (III) deposition in the brain has been reported [18], with evidence of the association between administration of GBCA and high signal intensity on unenhanced T1-weighted images in the dentate nucleus and globus pallidus [18,19,20]. Accumulation of Gd (III) in the brain is mostly associated with exposure to linear GBCA; however, Gd (III) brain deposition resulting from use of macrocyclic agents, although at lower levels, has also been documented [14]. Transmetallation, active metal transporters in cell membranes, and the lymphatic system have been pointed out as alternative access routes to the brain, contributing to Gd (III) brain deposition [14].

Several potential mechanisms of Gd (III) cytotoxicity have been pointed out, namely upregulation of inflammation, oxidative stress, apoptotic and profibrotic mechanisms [21]; moreover, Gd (III) seems to interfere with iron mobilization [7,22], as shown by the associated alterations in iron-binding capacity, serum iron, ferritin and transferrin saturation (TSAT) [23,24,25]. Iron involvement in Gd (III) toxicity also aligns with the transmetallation process; additionally, gadodiamide, a linear GBCA, was found to induce differentiation of peripheral blood mononuclear cells into ferroportin-expressing fibrocyte-like cells, facilitating Gd (III) efflux, but causing disturbances in iron homeostasis [26]. In mice with NSF, gadodiamide caused the release of catalytic iron that was prevented by the iron chelator deferiprone [7]. The transmembrane divalent metal transporter 1 (DMT1), present in the cell membrane of the intestine, kidney, brain and other cell tissues, mediates iron uptake into cells, and it seems to also mediate the transport of other divalent metals, such as manganese, zinc, cobalt and copper. In dietary iron overload conditions, DMT1 expression was found to be downregulated, while iron deficiency appears to favor the absorption of other metals [27,28].

The amount of Gd (III) that may be released after exposure to GBCA, and to what extent Gd (III) is accumulated in different tissues, remains incompletely clarified, as does its clinical significance, particularly regarding its potential impact on the homeostasis and metabolism of endogenous divalent metals. With the purpose of contributing to clarifying this knowledge gap, we evaluated in healthy Wistar rats the extent of renal and brain levels of Gd (III), iron, calcium, zinc, copper and manganese, 2 days after exposure to a single dose of Gd-DOTA or to free Gd (III); the retention of Gd (III) in these organs and its release into the bloodstream in the long term (20 weeks after exposure) and the implications of the exposure to Gd-DOTA or to free Gd (III), over short- and long-term periods after exposure, on kidney and brain iron deposition and on iron metabolism were also evaluated.

## 2. Results

In a previous study from our group [12], mild glomerular lesions were already observed 2 days after exposure to free Gd (III), whereas advanced tubulointerstitial lesions and altered renal function biomarkers were only evident after 20 weeks. In contrast, no significant changes were observed following Gd-DOTA administration at either time point.

**Figure 1:**
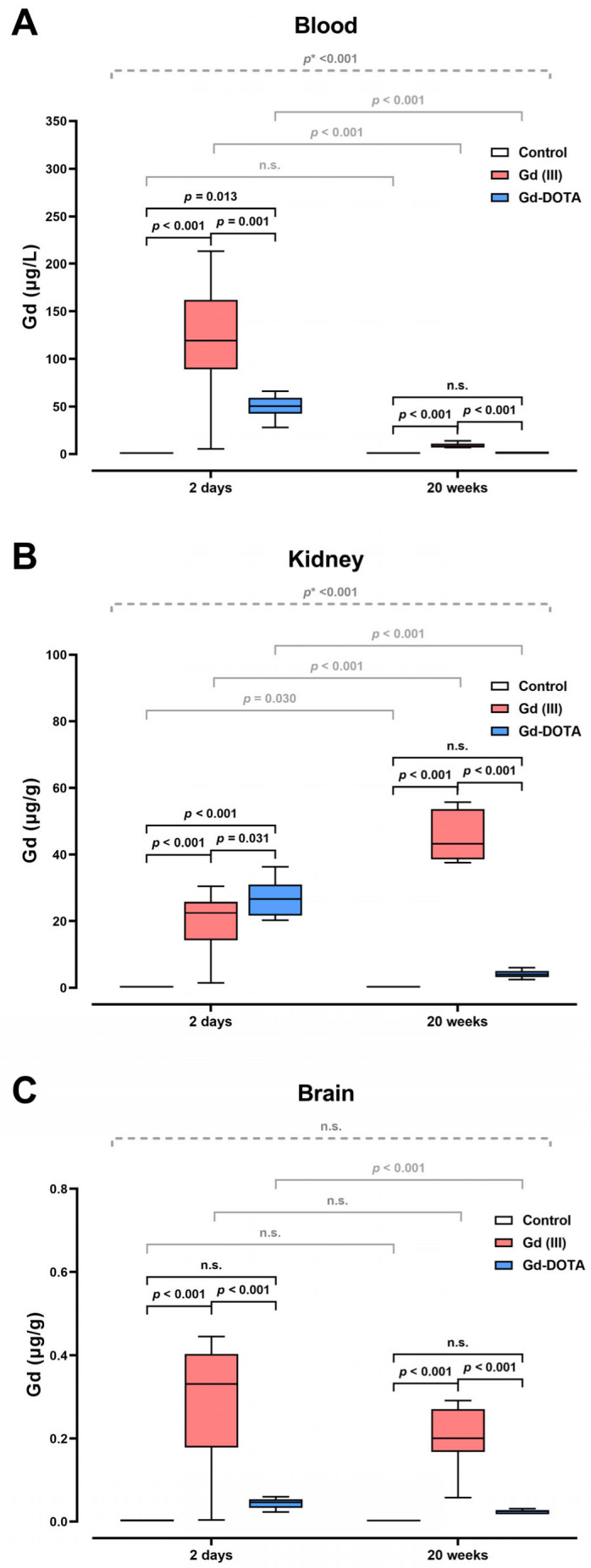
Gadolinium [Gd (III)] levels quantified in (**A**) blood, (**B**) kidney and (**C**) brain, 2 days and 20 weeks after exposure to a single dose of free Gd (III) or of gadoteric acid (Gd-DOTA). Data presented as median (inter-quartile range) (boxes) and maximum/minimum (whiskers) values of at least 7 animals/group. Black brackets—one-way ANOVA or Kruskal–Wallis H test (both followed by Bonferroni post hoc Test) between groups within each timepoint; gray brackets—Student’s *t*-test or Mann–Whitney U test between time points within each treatment group; gray dashed brackets—two-way ANOVA test for analysis of time × treatment interaction (*p*-value represented by p*); *p* < 0.05 was considered statistically significant; n.s., non-significant.

Blood Gd (III) levels (Figure 1A) were significantly higher compared to controls, both 2 days and 20 weeks after exposure to free Gd (III), although a significant decrease over time was observed.

Following Gd-DOTA exposure, blood Gd (III) concentrations were also elevated after 2 days, but significantly lower than those observed with free Gd (III); after 20 weeks, the levels were similar to controls, and significantly lower than those observed after exposure to free Gd (III). As with free Gd (III), blood levels decreased significantly over time after exposure to Gd-DOTA.

Regarding tissue retention, kidney Gd (III) levels were significantly increased 2 days after exposure to free Gd (III), and further increased at 20 weeks (Figure 1B). After Gd-DOTA exposure, kidney Gd (III) levels were also significantly higher than the controls at 2 days; 20 weeks after exposure, levels decreased, but still remained above control levels and were significantly lower than those observed after exposure to free Gd (III). Overall, renal Gd (III) deposition increased over time following exposure to free Gd (III), whereas it decreased over time following Gd-DOTA exposure.

At both time points after exposure to free Gd (III), brain Gd (III) deposition (Figure 1C) was significantly higher than in controls, with no significant changes over time. In contrast, in the Gd-DOTA group, brain Gd (III) values did not differ significantly from controls, and were significantly lower than those observed after Gd (III) exposure at both time points; a decrease over time was also observed.

**Figure 2:**
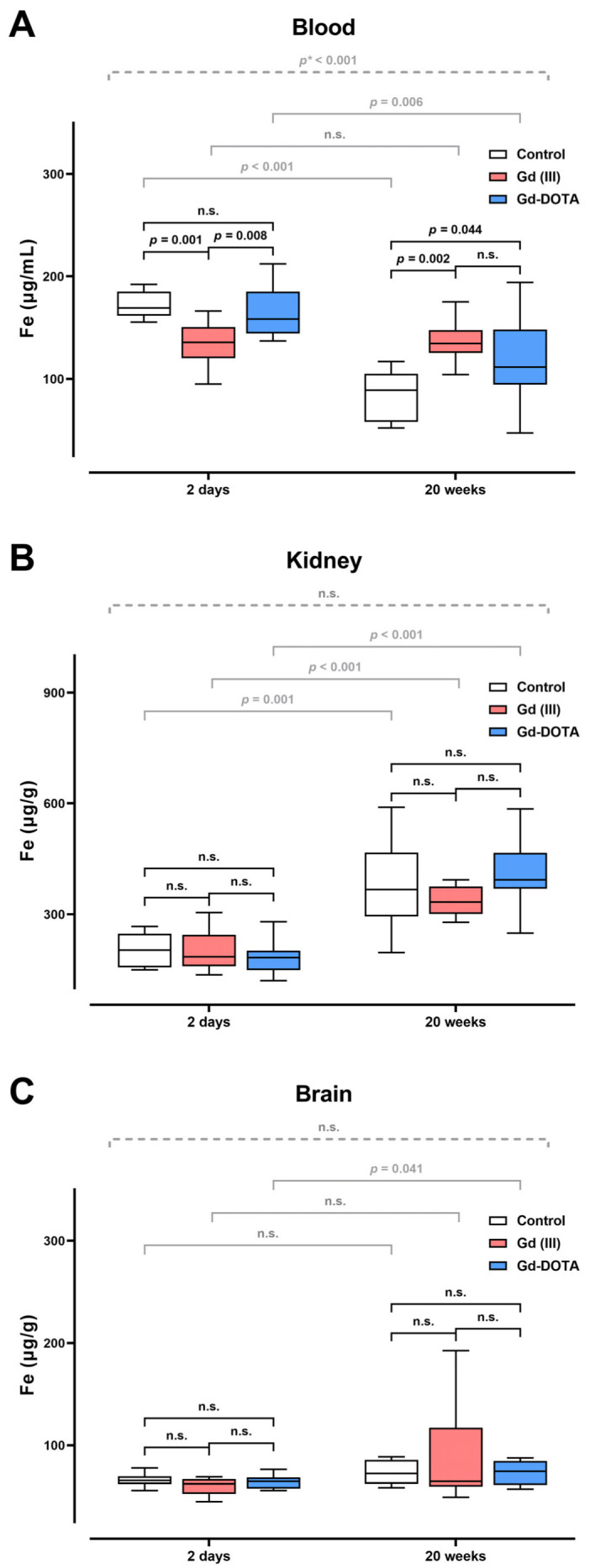
Iron (Fe) levels quantified in (**A**) blood (serum), (**B**) kidney and (**C**) brain, 2 days and 20 weeks after exposure to a single dose of free gadolinium [Gd (III)] or gadoteric acid (Gd-DOTA). Data presented as median (inter-quartile range) (boxes) and maximum/minimum (whiskers) values of at least 7 animals/group. Black brackets—one-way ANOVA or Kruskal–Wallis H test (both followed by Bonferroni post hoc Test) between groups within each timepoint; gray brackets—Student’s *t*-test or Mann–Whitney U test between time points within each treatment group; gray dashed brackets—two-way ANOVA test for analysis of time × treatment interaction (*p*-value represented by p*); *p* < 0.05 was considered statistically significant; n.s., non-significant.

Compared to the control, circulating iron levels (Figure 2A) were significantly lower 2 days after exposure to free Gd (III), but significantly higher at 20 weeks.

In the Gd-DOTA group (Figure 2A), iron levels were similar to controls after 2 days, and significantly higher than those found after exposure to free Gd (III); at 20 weeks, iron levels were significantly higher than controls. Of note, iron levels decreased over time both in the control and Gd-DOTA-treated groups, whereas no similar reduction was observed following exposure to free Gd (III).

In contrast to the circulating levels, kidney and brain iron (Figure 2B,C) did not differ significantly among the three groups studied, at either time points. A significant increase in kidney iron values was observed between 2 days and 20 weeks after exposure to free Gd (III) or to Gd-DOTA, and also for the control group (Figure 2B). Concerning brain iron levels (Figure 2C), no statistically significant difference over time was observed, except for animals exposed to Gd-DOTA that showed significantly higher levels at 20 weeks compared to 2 days after exposure.

In the Gd (III) group, 2 days after exposure, ferritin levels (Table 1) were significantly increased, whereas TSAT values were significantly decreased compared to the control. At 20 weeks, the opposite pattern was observed, with lower ferritin and higher TSAT values.

In controls, ferritin increased and TSAT decreased over time, while the reverse trend was observed after exposure to free Gd (III). In the Gd-DOTA group, no significant changes were observed in ferritin or TSAT at either time point. Compared to Gd-DOTA, exposure to free Gd (III) resulted in higher ferritin at 2 days, and lower ferritin at 20 weeks, while TSAT was significantly lower 2 days after exposure to free Gd (III).

The blood zinc levels increased over time after exposure to free Gd (III) or Gd-DOTA, and were significantly higher in the kidneys 20 weeks after free Gd (III) exposure, compared with controls (Table 1).

Two days after exposure to free Gd (III), blood calcium levels were significantly higher than in the other two groups; kidney calcium content increased over time and, at 20 weeks, was significantly higher than in controls, whereas brain calcium levels decreased from 2 days to 20 weeks, remaining significantly lower than those of the control group (Table 1).

Regarding manganese blood levels (Table 1) 2 days after exposure to free Gd (III), they were significantly higher than in the control group, whereas 20 weeks after exposure to Gd-DOTA, brain manganese levels were significantly lower than those observed in controls.

Blood and brain copper levels increased over time after exposure to free Gd (III) or Gd-DOTA; 2 days after exposure to free Gd (III), blood copper levels were significantly higher than in controls and after exposure to Gd-DOTA; long-term kidney copper content was significantly higher than in controls and increased significantly over time in the controls, Gd (III) and Gd-DOTA groups (Table 1).

**Figure 3:**
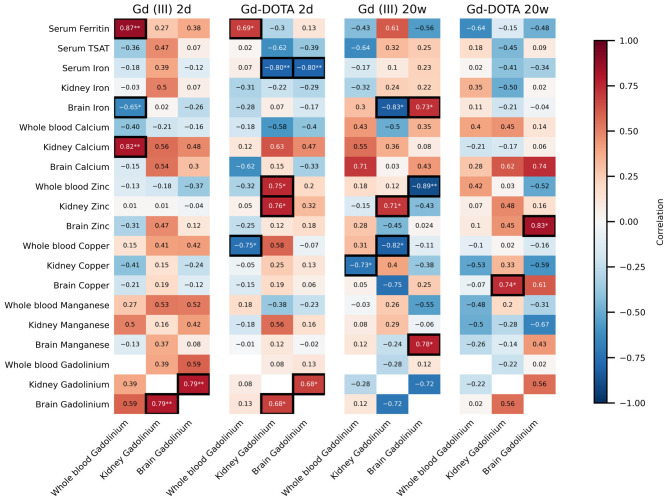
Heatmaps of Spearman correlation coefficients between gadolinium [Gd (III)] levels in whole blood, kidney, and brain and ferritin, transferrin saturation (TSAT) and systemic and tissue metal levels for Gd (III) and gadoteric acid (Gd-DOTA) groups at 2 days (2 d) and 20 weeks (20 w). Correlation coefficients are displayed within each cell. Significant correlations (* *p* < 0.05, ** *p* < 0.01) are indicated by asterisks and highlighted with black borders. Color scale represents correlation strength and direction (blue = negative, red = positive).

Overall, correlations between Gd (III) and metal levels were predominantly observed at 2 days after exposure (Figure 3), and included positive associations between blood Gd (III) and ferritin, inverse relationships with iron parameters, and positive correlations between kidney and brain Gd (III) levels in both Gd (III) and Gd-DOTA groups. At 20 weeks after exposure to free Gd (III), brain iron values were negatively correlated with kidney Gd (III) levels, while a positive correlation was found with brain Gd (III) values. Calcium showed limited association with Gd (III) levels, with only kidney calcium showing a positive correlation with blood Gd (III) 2 days after exposure to free Gd (III). Significant correlations (Figure 3) were more evident following Gd-DOTA exposure over the short term, particularly involving zinc and copper; long-term exposure to free Gd (III) was characterized by a limited number of tissue-specific associations with iron, zinc, manganese, and copper.

## 3. Discussion

As far as we know, no other study has evaluated, in healthy rats, the levels of Gd (III) and key endogenous metals involved in transmetallation, as well as their relationships, in the kidney, brain, and blood after a single-dose exposure to Gd-DOTA or to free Gd (III), both over short- (2 days) and long-term (20 weeks) periods.

Linear GBCA are more prone than macrocyclic GBCA to release Gd (III), which may accumulate in several organs [17]. Different studies using animal models, either healthy or presenting infectious or inflammatory conditions, showed that exposure to linear GBCA induced Gd (III) deposition at several tissues/organs, such as the skin, bone, liver, and brain [29,30]. Macrocyclic GBCA, although considered safer, has also been associated with deposition of Gd (III) in several tissues/organs. Pharmacodynamic and pharmacokinetic properties presented by the macrocyclic agents seem to influence Gd (III) retention. In fact, a similar exposure to macrocyclic GBCA may show different levels of Gd (III) deposition in the same tissues/organs, such as the cerebellum [31,32,33], cerebrum [33], kidney [31,33,34,35,36], liver [29,31,33,34,36,37], bone [29,30,31,33,34], spleen [34,36], skin [29,31,33,36,37] and lung [36]; moreover, it was also shown that after exposure to macrocyclic GBCA, the Gd (III) clearance from tissues/organs may be different [38]. The total body amount of Gd measured over a 5-month period, following gadopiclenol exposure in healthy animals, was 25–40% lower than that observed after exposure to Gd-DOTA or gadobutrol [13]. A study by Kartamihardja et al. showed that intact GBCA may pass through brain barriers, but their penetration ability seems to depend on the chemical structure of GBCA, and their retention in the brain may be associated with the binding of Gd (III) with organic molecules, including proteins [39]. Data are often controversial, which is probably related to differences in experimental protocols, namely doses and number of exposures, wash-out periods, and time after exposure when the analytical evaluations were carried out.

Human studies evaluating Gd (III) deposition after exposure to GBCA in patients with normal renal function are scarce. A study by Layne et al. in patients without renal dysfunction, exposed to a median of 2 exposures to GBCA, reported that 5 months (approximately 20 weeks) after the last exposure, Gd (III) was detected in 69.2%, 78.6%, and 95.2% of whole blood, plasma and urine samples, respectively [40]; accordingly, the authors suggested that further studies are needed to explore pharmacokinetics and pharmacodynamics of GBCA and the clinical significance of these detectable Gd (III) concentrations in patients with normal renal function. Even a single exposure to GBCA, in healthy conditions, seems to favor Gd (III) retention.

Our aim was, actually, to contribute to clarifying the impact of a single exposure to Gd-DOTA, a GBCA commonly used in MRI studies, and to free Gd (III), in the absence of renal dysfunction, by evaluating Gd (III) and endogenous metal values in blood, kidney and brain tissues, over short- (2 days) and a long-term (20 weeks) periods after exposure. Given that transmetallation with iron has been proposed as a key mechanism of toxicity for GBCA injury and the fact that this may alter iron homeostasis, we also evaluated the blood levels of iron, ferritin and TSAT.

We found that 2 days and 20 weeks after exposure to a single dose of the compounds, Gd (III) was detectable in blood, kidney and brain samples, for both Gd (III) and Gd-DOTA groups. The tissue Gd (III) values were higher for animals exposed to free Gd (III) than to Gd-DOTA, except in the case of the kidney samples, 2 days after administration.

For the two compounds, we found the lowest values of Gd (III) in the brain, which is in accordance with Kartamihardja et al.’s study, reporting a small amount of Gd (III) in the brain after Gd-DOTA exposure [39]. It was reported that in healthy and renal failure mouse models repeatedly exposed to Gd-DOTA or gadodiamide, Gd (III) was eliminated from the brain 45 days after the last exposure to Gd-DOTA, in the cases of both normal or altered renal function, while for the linear GBCA, Gd (III) clearance was limited [32]. We evaluated brain Gd (III) levels at 20 weeks after the exposure to Gd-DOTA, and very small amounts of Gd (III) were still detectable, although not significant, as compared to the controls.

Two days after exposure to free Gd (III), the kidney tissue showed significantly higher Gd (III) levels than brain tissue and their values correlated positively, suggesting the co-occurrence of both deposition and excretion; 20 weeks after exposure to free Gd (III), the levels of Gd (III) in the blood and brain were lower than those observed 2 days after exposure, but were higher in the kidney.

Our data also showed that 2 days after exposure to free Gd (III), the circulating levels of Gd (III), as compared to Gd-DOTA, were significantly increased; however, at the same time point, the Gd (III) values in kidney tissue were significantly higher after Gd-DOTA exposure than after free Gd (III) exposure, suggesting a more efficient excretion for Gd-DOTA; this may explain the significantly lower levels of Gd (III) retained in the brain, as compared to its value after exposure to Gd (III) (almost sixfold higher). The apparent more efficient renal excretion of Gd-DOTA seems to be associated with a lower toxicity, by reducing the deposition of Gd (III) in brain tissue and, possibly, in other tissues. In line with this, the Gd (III) blood levels 20 weeks following exposure to Gd-DOTA were similar to those of the controls, suggesting an almost complete excretion of Gd (III), while in the case of free Gd (III) exposure, the Gd (III) blood values were still significantly higher than those of the controls, and also significantly higher than those of the Gd-DOTA group. Indeed, the slower renal excretion of Gd (III) after exposure to free Gd (III) may explain the lower amount of Gd (III) in the kidney, alongside higher values of Gd (III) in the brain, after 2 days of exposure. It seems that the lower excretion of Gd (III) favors its retention in blood and tissues, observed 2 days after exposure to free Gd (III), and explains the higher Gd (III) values in the blood, brain, and kidney, 20 weeks after Gd (III) exposure. The longer exposure of renal cells or of other body cells, to (not-excreted) Gd (III) may lead to structural and/or dysfunctional cellular changes. This is in accordance with our previous report [12], in which, by performing renal histopathological studies to evaluate kidney injuries, over short- and long-term after exposure to Gd (III) or to Gd-DOTA, we observed that 20 weeks after exposure to free Gd (III) the animals presented higher scores of mild glomerular lesions and advanced tubulointerstitial lesions, as compared to controls and to animals exposed to Gd-DOTA.

The kidney is a preferential target for Gd (III) deposition and injury, given its main role in Gd (III) excretion, although its excretion and/or deposition depend on the pharmacodynamic and pharmacokinetic properties of each GBCA [41]. Accordingly, a study in nephrectomized animals administered with two doses of different GBCA showed different degrees of Gd (III) deposition in studied tissues, but predominantly deposition occured in the kidney for all studied GBCA [36]. Also in accordance with this, another study showed that after a single administration of Gd (III), gadodiamide or gadobutrol, high levels of Gd (III) in the kidney and skin were a common finding for all compounds [22].

Di Gregorio et al. [34] reported that Gd (III) values decreased over time since the last exposure to gadodiamide or gadoteridol in healthy animals. Another study, with Wistar rats repeatedly exposed to different GBCA, showed that the kidney cortex exhibited higher Gd (III) retention by the 17th day, decreasing by the 34th and 52nd days following administration [35]. After repeated exposures to GBCA, the highest kidney and cerebellum Gd (III) levels were found in the 6th week, followed by a significant decrease in the 10th week [31]. Accordingly, we also observed that the kidney’s Gd (III) concentrations decreased over time following exposure, from 2 days to 20 weeks of administration of Gd-DOTA.

Concerning kidney iron levels, we found an increase over time (with aging) in controls, and in the Gd (III) and Gd-DOTA groups, and no significant changes between the three groups at both time points, suggesting no significant changes in renal iron deposition.

The brain iron amount did not differ in controls between 2 days and 20 weeks, and the same was found for the Gd (III) group; for the Gd-DOTA group, a significant increase was observed, although, at this time point (20 weeks after exposure), the median iron values were similar to those values presented by the control and Gd (III) groups.

Regarding iron, TSAT and ferritin circulating values, in controls, the first two decreased significantly over time, while ferritin increased. Compared to controls, after 2 days of exposure to Gd (III), the animals presented significantly lower iron and TSAT, and significantly higher ferritin values, suggesting a disturbance in iron homeostasis induced by Gd (III); a trend for the same changes was found at 2 days after exposure to Gd-DOTA that may be explained by the faster elimination of Gd (III) and, therefore, a lower interaction with iron homeostasis.

It has been proposed that GBCA, by targeting iron-recycling and ferroportin-expressing macrophages, triggers iron mobilization from ferritin stores, contributing with labile iron that can be used in GBCA transmetallation and increase TSAT [24]; these changes were only observed 2 days after exposure to free Gd (III), suggesting an association with GBCA transmetallation; nevertheless, given that the exposure to free Gd (III) was shown to trigger inflammation and tissue injuries [12], the changes observed in iron metabolism 2 days after exposure to free Gd (III) may also reflect the well-known disturbances associated with inflammation in iron homeostasis, namely an increase in ferritin, a reduction in iron, and unchanged or slightly lower TSAT. As already reported [12], the exposure to Gd-DOTA did not induce a significant inflammatory response in the short-term, supporting this hypothesis. In fact, we found mild changes in blood biomarkers and histological changes associated with an inflammatory milieu at the same time point.

A single exposure to free Gd (III) or Gd-DOTA appeared to have no significant impact on kidney and brain iron deposits; however, exposure to free Gd (III) seems to interfere with iron metabolism by triggering an inflammatory stimulus and/or by targeting iron-recycling macrophages, at 2 days after exposure. In line with this, we found that, at this time point, ferritin levels correlated positively with Gd (III) blood concentrations.

At 20 weeks after exposure to free Gd (III), the Gd (III) blood levels were similar to the controls, ferritin was significantly lower, and iron and TSAT were significantly increased, suggesting that the inflammatory stimulus was already residual and further supporting the contribution of inflammation to the observed changes in iron homeostasis in the short term.

In the case of exposure to Gd-DOTA, comparing the two evaluated time points, the changes are consistent with a lower Gd (III)-induced inflammatory state, presenting residual changes in iron homeostasis. Of note, 2 days after exposure to free Gd (III), the brain iron values were significantly and negatively correlated with Gd (III) blood levels, further suggesting that, although residual, transmetallation may occur. We cannot rule out the hypothesis that Gd (III) also interferes with tissue iron deposition in other organs, since iron body stores are primarily in the liver, spleen, bone marrow, and skeletal muscles. In fact, it has been reported that exposure to Gd (III) caused enlargement of the spleen, probably due to deposition of Gd (III) or iron [22]. One the other hand, the observed alterations in circulating iron-related biomarkers, despite the absence of measurable changes in tissue iron content, may reflect a dysregulation of systemic iron homeostasis rather than net iron accumulation within tissues. There is a clear need for mechanistic studies to further clarify the relationship between Gd (III) retention and alterations in iron, as well as in other endogenous metal, homeostasis.

Overall, the correlation patterns highlight distinct relationships between Gd (III) distribution and systemic or tissue metal homeostasis, which vary depending on both the chemical form and time after exposure. Importantly, the temporal evolution of these correlations differed markedly between Gd (III) and Gd-DOTA, pointing to distinct biological behaviors for the two forms. While Gd-DOTA exhibited widespread correlations in the short term that diminished over time, Gd (III) showed a progressive increase in significant predominantly tissue-specific associations at long term, suggesting divergent temporal dynamics.

After 2 days of exposure to either free Gd (III) or Gd-DOTA, significant positive correlations between kidney and brain Gd (III) levels indicate a coupled distribution between these compartments. This supports the hypothesis that in the short term, Gd (III) follows a systemic distribution pattern, likely reflecting ongoing circulation and exchange of organ retention, as demonstrated in previous studies [4,35].

Calcium, particularly in kidney tissue, showed a strong positive correlation with circulating Gd (III) levels in the Gd (III) group. Given the chemical similarity between Gd (III) and calcium ions, these findings may reflect competition for binding sites or shared transport pathways, supporting the hypothesis that Gd (III) can interfere with calcium-dependent physiological processes [1].

Trace elements such as zinc, manganese, and copper exhibited variable and condition-dependent correlations. Some significant associations, particularly in the long term, may reflect adaptive or compensatory responses to long-lasting exposure and disruption of metal homeostasis [21]. The heterogeneity observed across tissues further suggests that these interactions are shaped by local metabolic environments rather than systemic regulation alone.

A higher number of significant correlations was observed in the Gd-DOTA group at 2 days after exposure, compared to the Gd (III) group, reflecting a broader pattern of early associations with multiple metal-related parameters. These correlations involve several elements, including iron, calcium, zinc, copper, and manganese, indicating that Gd (III), particularly in its chelated form, initially participates in a wide network of systemic and tissue metal interactions. Over time, this pattern diverges between compounds: while correlations in the Gd-DOTA group decrease, those in the Gd (III) group increase and become more tissue-specific. This divergence is most likely related to differences in stability and consequent availability of free Gd (III).

Notably, a consistent pattern was observed across experimental groups in which tissue-to-tissue correlations were predominantly positive, whereas circulating-to-tissue correlations were largely negative. This suggests that Gd (III) distribution follows compartment-specific dynamics, with coordinated retention within tissues but divergence from circulating levels. Such behavior is indicative of a shift from systemic distribution to localized accumulation, accompanied by reduced exchange between compartments. Importantly, this compartmental divergence aligns with disruptions in metal homeostasis. The inverse relationship between circulating and tissue levels may reflect competitive or compensatory mechanisms whereby Gd (III) disturbs the balance of endogenous metals across compartments. Given that essential metals, such as iron, copper and zinc, are tightly regulated through interconnected transport, storage, and redistribution pathways, the observed correlations suggest that Gd (III) exposure disrupts these regulatory networks. It appears that the increase in Gd (III) in tissues may result from changes in circulating metal levels, reflecting broader systemic adjustments aimed at maintaining metal balance under conditions of long-lasting exposure.

One major limitation of our study is that the method used to quantify metals does not allow us to distinguish whether measured Gd (III) is present in a chelated or unchelated state; the reported values reflect total Gd (III) value rather than its chemical speciation. Although Gd-DOTA exhibits high thermodynamic stability and kinetic inertness, it cannot be excluded that a small fraction of Gd (III) may be released from the GBCA, particularly during long-term retention, which could potentially contribute to the biological effects observed herein and previously reported [12]. Another limitation of our study may be the limited number of animals per group that may reduce the power of the generated data, despite following a power analysis performed to establish minimal animal group size. Also, the pharmacokinetic and toxicokinetic profiles of the compounds may differ between animals and humans. Because the present study was restricted to the analysis of kidney and brain tissues, we were unable to ascertain whether iron and other metals underwent redistribution to other organs, including the liver and spleen. We evaluated the effects of a single dose of Gd-DOTA in healthy animals at both short- and long-term time points, with particular emphasis on Gd (III) deposition in the kidneys and brain; still, the investigation of the impact of repeated exposure to different macrocyclic GBCA across multiple organs and at various time points, to better characterize the kinetic profile of Gd (III) distribution and elimination, not only in healthy subjects but also in experimental models representing vulnerable conditions, is warranted. Our data highlight the need to determine the levels of each type of GBCA inducing physiopathological changes; and to clarify the short- and long-term implications of Gd (III) retention and/or tissue accumulation. The present findings are limited to a healthy animal model and cannot be directly extrapolated to patients with renal impairment; however, individuals with compromised renal function may present altered Gd (III) clearance, potentially leading to increased retention and different biological effects, which warrant further investigation in relevant disease models. Therefore, we propose that studies monitoring circulating Gd (III) levels, in the case of repeated exposure to GBCA and in cases of renal insufficiency, should be performed, as Gd (III) toxicity may be potentiated under these conditions. In addition, the effects of repeated exposure to Gd-DOTA, particularly in cases of mild renal disease, often undiagnosed, deserve further studies.

### Conclusion Remarks

In summary, in healthy animals, a single exposure to Gd-DOTA or free Gd (III) led to persistent, detectable Gd (III) in blood, kidney, and brain tissues, for up to 20 weeks, with higher tissue retention after exposure to Gd (III). The kidney was the main target, while brain levels remained low but measurable over time. Gd (III) also appeared to affect iron, copper, calcium and zinc homeostasis. These results support the need for further studies on long-term tissue retention, metal–gadolinium interactions, and the clinical relevance of low-level persistent Gd (III), particularly under repeated exposure and/or impaired renal function.

## 4. Materials and Methods

### 4.1. Animal Experimental Protocol

For the experimental protocol, we used eight-week-old male Wistar rats, purchased from Charles River Laboratories, Barcelona, Spain. Animals were housed in ventilated cages, under controlled temperature (22 °C) and humidity (50–60%), exposed to 12 h dark/light cycles, with free access to tap water and rat laboratory chow (4RF21 Mucedola, Milan, Italy).

The project was approved (#7-2022, on 28 November 2022) by the local (iCBR-FMUC) Animal Welfare Body (ORBEA) and the Portuguese Authority (DGAV). All animal procedures were carried out in accordance with the National (DL 113/2013) and European (2010/63/EU) Animal Care Directives, and the report followed the ARRIVE guidelines [42].

The rats were randomly divided into six groups (*n* = 10/group); a power analysis was performed to establish the animal group size and no exclusion criteria were defined. To minimize subjective bias, a blinded experimental design was implemented. Group allocation was performed by an independent researcher. Researchers involved in administering treatments and conducting experiments were aware of group identities; however, outcome assessment and the data analysis were performed by researchers who were kept completely blind to group allocation throughout the study. The allocation code was only broken after all data analysis was complete. In both the short- and long-term studies, 3 sets of animals were exposed to a single dose of: Gd (III) (in the salt form of GdCl_3_·6H_2_O; 0.1 mmol/kg); Gd-DOTA (0.1 mmol/kg); or a vehicle (saline; control group), by tail vein injection. The selected Gd-DOTA dose corresponds to a single administration used in humans for MRI studies. Both Gd (III) and Gd-DOTA injectable solutions were freshly prepared on the day of exposure in sterile 0.9% (*m*/*v*) saline, at a concentration of 0.08 mmol/mL, ensuring complete solubilization. The solutions were prepared under aseptic conditions, at physiological pH (approximately 7.4), and used immediately after preparation to minimize potential changes in chemical stability. Prior to administration, animals were individually weighed, and the injection volume was adjusted to 1.25-times the animal’s body weight (mL/kg), in order to achieve the target dose of 0.1 mmol/kg. This approach also ensured that the injected volume remained below 500 µL, thus complying with recommended limits for rapid bolus tail vein injection. The rats were under volatile anesthesia with isoflurane: 2–3% of Isoflurin^®^ (Dechra, Boston, MA, USA) in 100% oxygen. The protocol lasted until 2 days or 20 weeks after administration of the compounds or vehicle, for evaluation of the short- or long-term effects, respectively.

### 4.2. Sample Collection

At the end of the protocol, animals were sacrificed using an overdose (80 mg/kg) of intraperitoneal pentobarbital (Sigma-Aldrich, Saint Louis, MO, USA). Blood was collected from the left ventricle, by cardiac puncture, into tubes without and with anticoagulant (K_3_EDTA) to obtain serum or whole blood, respectively. After perfusion with saline solution, the right kidney and brain were removed, cleaned, and processed for Gd (III) and iron evaluations. Aliquots of serum, blood, kidney and brain were immediately stored at −20 °C until assayed.

### 4.3. Assays

Using routine automated methods (Siemens Healthineers, Erlangen, Germany), we evaluated serum levels of iron and TSAT; we also measured ferritin, by immunoturbidimetry (Siemens Healthineers, Erlangen, Germany).

Deposition of Gd (III), iron, calcium, zinc, copper and manganese in the kidney and brain, as well as their blood levels (except for iron), were assessed by inductively coupled plasma mass spectrometry (ICP-MS) using an iCAP™ Q instrument (Thermo Fisher Scientific, Bremen, Germany). The system was equipped with a TQ+ quartz concentric nebulizer (Meinhard, Golden, CO, USA), a Peltier-cooled high-purity quartz baffled cyclonic spray chamber and a demountable quartz torch with a 2.5 mm i.d. quartz injector, and a CETAC ASX-520 auto-sampler (Teledyne CETAC Technologies, Omaha, NE, USA). The instrument was tuned for maximum sensitivity, signal stability, and minimal formation of oxides and double-charged ions [typical operating conditions included nebulizer, auxiliary, and plasma gas flows of 1.08, 0.79, and 13.9 L/min, respectively; a radio frequency power of 1550 W; and a dwell time of 10–100 ms] [43]. Prior to ICP-MS analysis, tissue samples were dried by incubation at 60 °C, until constant weight, which was recorded. Subsequently, kidney, brain, and blood samples were digested using a mixture of 65% (*w*/*w*) HNO_3_ and 30% (*v*/*v*) H_2_O_2_, followed by incubation at room temperature for 2–3 days. For analytical quality control, certified reference materials BCR-670 and ERM-CE278k (Institute for Reference Materials and Measurements, European Commission–Joint Research Centre, Geel, Belgium) as well as Seronorm™ whole blood L1 quality control sample (Sero AS, Billingstad, Norway) spiked with different concentrations of Gd (III) were digested and analyzed alongside the samples.

### 4.4. Statistical Analysis

The IBM Statistical Package for Social Sciences (SPSS) for Windows, version 29.0 (IBM, Armonk, NY, USA) was used. Tukey’s Test was applied to identify outliers. Within each dataset, a maximum of three outliers were detected and excluded prior to statistical analysis. Therefore, results are from at least 7 animals/group. Shapiro–Wilk analysis was employed to verify if data were normally distributed. Differences between groups within each time point were evaluated by one-way ANOVA or Kruskal–Wallis H test, depending on whether the data followed a normal distribution or not; each was followed by the Bonferroni post hoc Test; for comparisons between time points within each treatment group, Student’s t-test or Mann–Whitney U test was used, depending on the distribution of data; two-way ANOVA was performed to examine the combined effect of both time and treatment. Spearman correlation coefficients were determined to explore the (cor)relation between parameters. Results are presented as mean ± standard error of the mean (SEM) or as median (inter-quartile range), according to data distribution. Statistical significance was accepted at *p* < 0.05.

## Figures and Tables

**Table 1:** Ferritin and transferrin saturation (TSAT) serum levels, and amount of zinc (Zn), calcium (Ca), manganese (Mn) and copper (Cu) in blood, kidney and brain, 2 days (2 d) and 20 weeks (20 w) after exposure to a single dose of free gadolinium [Gd (III)] or gadoteric acid (Gd-DOTA).

	Time Points	Control	*p*-Value	Gd (III) (0.1 mmol/kg)	*p*-Value	*p*-Value vs. Control	Gd-DOTA (0.1 mmol/kg)	*p*-Value	*p*-Value vs. Control	*p*-Value vs. Gd (III)	Time × Treatment Interaction, *p*
**SERUM**											
**Ferritin** (μg/dL)	2 d	30.2 ± 4.0	**<0.001**	138.9 ± 22.4	**0.012**	**<0.001**	48.6 ± 4.5	**0.009**	1.0	**<0.001**	**<0.001**
	20 w	249.7 ± 17.3		68.3 ± 6.6		**<0.001**	180. 6 ± 38.7		0.180	**0.011**	
**TSAT** (%)	2 d	69.7 ± 7.4	**0.006**	36.2 ± 3.2	**0.002**	**0.001**	57.7 ± 5.6	0.435	0.449	**0.047**	**<0.001**
	20 w	42.9 ± 3.2		64.9 ± 7.0		**0.009**	52.4 ± 2.3		0.588	0.274	
**BLOOD**											
**Zn** (µg/L)	2 d	5135 ± 281	**0.012**	5752 ± 99	**0.011**	0.075	5557 ± 102	0.276	0.377	1.0	0.113
	20 w	6022 ± 64		6266 ± 153		0.578	5762 ± 151		0.499	0.031	
**Ca** (×10^3^ µg/L)	2 d	63.9 ± 0.76	0.051	67.4 ± 1.1	0.093	**0.041**	61.3 ± 0.77	0.056	0.212	**<0.001**	**0.010**
	20 w	67.4 ± 1.4		65.0 ± 0.8		0.414	64.3 ± 1.2		0.192	1.0	
**Mn** (µg/L)	2 d	7.95 ± 0.47	0.881	9.15 ± 0.20	0.452	**0.030**	8.76 ± 0.10	0.944	0.244	1.0	0.493
	20 w	8.04 ± 0.32		8.88 ± 0.30		0.338	8.79 ± 0.44		0.489	1.0	
**Cu** (µg/L)	2 d	652 ± 43	**<0.001**	1082 ± 47	**<0.001**	**<0.001**	757 ± 19	**0.023**	0.236	**<0.001**	**<0.001**
	20 w	879 ± 19		803 ± 9		0.087	852 ± 32		1.0	0.414	
**KIDNEY**											
**Zn/dry weight** (µg/g)	2 d	91.1 ± 3.7	0.559	99.8 ± 1.8	0.690	0.164	93.8 ± 3.3	0.948	1.0	0.522	0.849
	20 w	88.3 ± 2.9		98.6 ± 2.2		**0.015**	93.5 ± 1.9		0.406	0.420	
**Ca/dry weight** (µg/g)	2 d	461 (383–576)	1.0	450 (359–500)	**0.011**	0.773	446 (408–482)	0.077	0.856	0.921	**0.039**
	20 w	456 (397–482)		521 (482–559)		**0.003**	470 (460–497)		0.289	0.057	
**Mn/dry weight** (µg/g)	2 d	3.61 ± 0.18	0.705	3.73 ± 0.10	0.330	1.0	3.72 ± 0.20	0.494	1.0	1.0	0.836
	20 w	3.53 ± 0.11		3.90 ± 0.13		0.114	3.56 ± 0.12		1.0	0.170	
**Cu/dry weight** (µg/g)	2 d	35.6 ± 2.9	0.661	39.0 ± 3.0	**0.037**	1.0	40.2 ± 2.0	0.989	0.687	1.0	**0.025**
	20 w	33.8 ± 2.8		59.4 ± 8.5		**0.009**	40.1 ± 3.6		1.0	0.064	
**BRAIN**											
**Zn/dry weight** (µg/g)	2 d	54.3 (51.6–72.0)	0.112	59.1 (56.9–67.9)	0.082	0.586	57.3 (52.3–60.0)	0.369	1.0	0.383	0.536
	20 w	51.5 (48.0–61.3)		45.6 (40.6–73.6)		0.318	50.6 (45.3–81.9)		0.876	0.415	
**Ca/dry weight** (µg/g)	2 d	255 ± 12	0.073	250 ± 9	**<0.001**	1.0	226 ± 10	0.089	0.184	0.334	0.086
	20 w	223 ± 12		179 ± 5		**0.021**	200 ± 9		0.349	0.581	
**Mn/dry weight** (µg/g)	2 d	2.33 ± 0.09	0.082	2.08 ± 0.07	0.068	0.090	2.02 ± 0.08	0.323	**0.029**	1.0	**0.020**
	20 w	2.10 ± 0.08		2.56 ± 0.23		0.169	2.15 ± 0.10		1.0	0.319	
**Cu/dry weight** (µg/g)	2 d	10.7 ± 0.5	**0.002**	10.3 ± 0.3	**0.019**	1.0	9.76 ± 0.16	**0.006**	0.238	0.933	0.438
	20 w	12.9 ± 0.3		11.9 ± 0.5		0.615	12.6 ± 0.7		1.0	1.0	

## Data Availability

The original contributions presented in this study are included in the article. The raw data supporting the conclusions of this article will be made available by the authors upon request. Further inquiries can be directed at the corresponding authors.

## Abbreviations

The following abbreviations are used in this manuscript:

DMT1	Divalent metal transporter 1
GBCA	Gadolinium-based contrast agents
Gd (III)	Gadolinium
Gd-DOTA	Gadoteric acid
ICP-MS	Inductively coupled plasma mass spectrometry
MRI	Magnetic resonance imaging
NSF	Nephrogenic systemic fibrosis
TSAT	transferrin saturation
